# Supplementary material for: Transcriptome and Differentially Expressed Gene Profiles in Mycelium, Primordium and Fruiting Body Development in Stropharia rugosoannulata
Source: Genes (Basel). 2022 Jun 17;13(6):1080. doi: 10.3390/genes13061080 (PMC9222559; doi:10.3390/genes13061080)
Supplement: Supplementary file 1 [file genes-13-01080-s001.zip › genes-1740782-Supplementary.pdf]

Supplementary Tables

Supplementary Table S1 Summary of the sequencing data of the *S. rugosoannulata* transcriptome at different growth stages.

Sample	Raw reads	clean reads	Total_Bases	Q20%	Q30%	GC%	mapped-reads	mapped-rate (%)
G-S-1	53243010	50516336	7.55E+09	98.28	94.37	54.48	49790461	94.75
G-S-2	81198788	76729644	1.15E+10	98.22	94.21	54.93	45041716	94.6
G-S-3	60501562	56967112	8.51E+09	98.33	94.51	54.54	47837984	93.35
P-S-1	67368950	63586950	9.5E+09	98.27	94.35	53.17	47647008	94.32
P-S-2	56544080	53295508	7.96E+09	98.3	94.4	53.6	71228128	92.83
P-S-3	57151492	54162064	8.09E+09	98.35	94.56	53.39	52210358	91.65
M-F-1	69871086	65825934	9.83E+09	98.2	94.16	53.48	59606406	93.74
M-F-2	56383966	53380648	7.98E+09	98.38	94.64	53.68	50337607	94.45
M-F-3	56731970	53847518	8.05E+09	98.39	94.67	53.54	50468211	93.18

Supplementary Table S2 GO functional classification of differentially expressed genes.

**G-S VS P-S**						
id	ratio_in_study	ratio_in_pop	p_uncorrected	p_fdr	description	namespace
GO:0019748	73/2969	153/11495	1.47E-06	0.004	secondary metabolic process	biological_process
GO:0000272	54/2969	107/11495	1.95E-06	0.004	polysaccharide catabolic process	biological_process
GO:0016798	80/2969	184/11495	2.27E-06	0.004	hydrolase activity, acting on glycosyl bonds	molecular_function
GO:0004553	78/2969	166/11495	2.50E-06	0.004	hydrolase activity, hydrolyzing O-glycosyl compounds	molecular_function
GO:0044550	62/2969	131/11495	2.62E-06	0.004	secondary metabolite biosynthetic process	biological_process
GO:0005576	103/2969	230/11495	2.79E-06	0.004	extracellular region	cellular_component
GO:0009404	34/2969	63/11495	3.40E-06	0.004	toxin metabolic process	biological_process
GO:0043386	28/2969	49/11495	3.62E-06	0.004	mycotoxin biosynthetic process	biological_process
GO:0044283	198/2969	567/11495	5.59E-06	0.006	small molecule biosynthetic process	biological_process
GO:0043385	28/2969	50/11495	5.80E-06	0.008	mycotoxin metabolic process	biological_process
GO:0044281	391/2969	1168/11495	6.18E-06	0.008	small molecule metabolic process	biological_process
GO:1901136	30/2969	55/11495	6.70E-06	0.008	carbohydrate derivative catabolic process	biological_process
GO:0016787	543/2969	1691/11495	7.26E-06	0.008	hydrolase activity	molecular_function
GO:1901135	174/2969	499/11495	8.15E-06	0.01	carbohydrate derivative metabolic process	biological_process
GO:0008152	1515/2969	5199/11495	9.31E-06	0.01	metabolic process	biological_process
GO:0017144	147/2969	412/11495	9.39E-06	0.01	drug metabolic process	biological_process
GO:0003824	1158/2969	3837/11495	9.41E-06	0.01	catalytic activity	molecular_function
GO:0003674	1746/2969	6261/11495	9.42E-06	0.01	molecular_function	molecular_function
GO:0071704	1384/2969	4817/11495	1.05E-05	0.01	organic substance metabolic process	biological_process

GO:0008150	1958/2969	6964/11495	1.05E-05	0.01	biological_process	biological_process
GO:0044237	1384/2969	4829/11495	1.06E-05	0.01	cellular metabolic process	biological_process
GO:0009403	29/2969	54/11495	1.32E-05	0.012	toxin biosynthetic process	biological_process
GO:0009987	1682/2969	6084/11495	1.33E-05	0.012	cellular process	biological_process
GO:1901378	24/2969	41/11495	1.38E-05	0.012	organic heteropentacyclic compound biosynthetic process	biological_process
GO:1901376	24/2969	41/11495	1.38E-05	0.012	organic heteropentacyclic compound metabolic process	biological_process
GO:1901657	39/2969	81/11495	1.69E-05	0.018	glycosyl compound metabolic process	biological_process
GO:1903825	51/2969	116/11495	2.41E-05	0.03	organic acid transmembrane transport	biological_process
GO:0044238	1246/2969	4443/11495	2.91E-05	0.04	primary metabolic process	biological_process
GO:0042180	32/2969	63/11495	3.10E-05	0.044	cellular ketone metabolic process	biological_process
GO:0016836	26/2969	48/11495	3.64E-05	0.046	hydro-lyase activity	molecular_function
**M-F VS P-S**						
GO:0015935	43/3287	63/11495	7.39E-07	0	small ribosomal subunit	cellular_component
GO:0022627	33/3287	48/11495	1.11E-06	0	cytosolic small ribosomal subunit	cellular_component
GO:0098800	48/3287	76/11495	1.21E-06	0.002	inner mitochondrial membrane protein complex	cellular_component
GO:0000313	34/3287	52/11495	1.26E-06	0.002	organellar ribosome	cellular_component
GO:0005761	34/3287	52/11495	1.26E-06	0.002	mitochondrial ribosome	cellular_component
GO:0015934	67/3287	113/11495	1.61E-06	0.002	large ribosomal subunit	cellular_component
GO:0022625	46/3287	78/11495	1.71E-06	0.002	cytosolic large ribosomal subunit	cellular_component
GO:0044391	109/3287	175/11495	1.75E-06	0.002	ribosomal subunit	cellular_component
GO:0098798	102/3287	195/11495	1.87E-06	0.002	mitochondrial protein complex	cellular_component
GO:0005840	119/3287	209/11495	1.94E-06	0.002	ribosome	cellular_component
GO:0140013	79/3287	164/11495	1.94E-06	0.002	meiotic nuclear division	biological_process

GO:0003735	100/3287	154/11495	2.20E-06	0.002	structural constituent of ribosome	molecular_function
GO:0061982	73/3287	154/11495	2.20E-06	0.002	meiosis I cell cycle process	biological_process
GO:0006261	93/3287	199/11495	2.42E-06	0.002	DNA-dependent DNA replication	biological_process
GO:0051785	59/3287	116/11495	2.42E-06	0.002	positive regulation of nuclear division	biological_process
GO:0006310	94/3287	207/11495	2.55E-06	0.002	DNA recombination	biological_process
GO:0022626	80/3287	139/11495	2.62E-06	0.002	cytosolic ribosome	cellular_component
GO:0007127	67/3287	139/11495	2.62E-06	0.002	meiosis I	biological_process
GO:0005759	109/3287	237/11495	2.86E-06	0.002	mitochondrial matrix	cellular_component
GO:0005198	165/3287	320/11495	3.03E-06	0.002	structural molecule activity	molecular_function
GO:1902600	39/3287	69/11495	3.05E-06	0.002	proton transmembrane transport	biological_process
GO:0000280	130/3287	286/11495	3.11E-06	0.002	nuclear division	biological_process
GO:0006839	124/3287	286/11495	3.11E-06	0.002	mitochondrial transport	biological_process
GO:0000819	91/3287	203/11495	3.11E-06	0.002	sister chromatid segregation	biological_process
GO:0048285	135/3287	303/11495	3.12E-06	0.002	organelle fission	biological_process
GO:0007059	126/3287	287/11495	3.13E-06	0.002	chromosome segregation	biological_process
GO:1990542	115/3287	255/11495	3.19E-06	0.002	mitochondrial transmembrane transport	biological_process
GO:0032543	51/3287	100/11495	3.22E-06	0.002	mitochondrial translation	biological_process
GO:0043604	214/3287	529/11495	3.36E-06	0.002	amide biosynthetic process	biological_process
GO:0006260	95/3287	205/11495	3.37E-06	0.002	DNA replication	biological_process
GO:0009100	69/3843	132/11495	9.93E-06	0.004	glycoprotein metabolic process	biological_process
**M-F VS G-S**						
GO:0004553	84/3434	166/11495	1.96E-06	0	hydrolase activity, hydrolyzing O-glycosyl compounds	molecular_function
GO:0005576	112/3434	230/11495	2.76E-06	0	extracellular region	cellular_component
GO:0006302	104/3434	232/11495	2.90E-06	0	double-strand break repair	biological_process

GO:0016798	93/3434	184/11495	2.92E-06	0	hydrolase activity, acting on glycosyl bonds	molecular_function
GO:0016054	65/3434	121/11495	2.98E-06	0	organic acid catabolic process	biological_process
GO:0046395	65/3434	121/11495	2.98E-06	0	carboxylic acid catabolic process	biological_process
GO:0007059	129/3434	287/11495	3.03E-06	0	chromosome segregation	biological_process
GO:0040020	39/3434	68/11495	3.11E-06	0	regulation of meiotic nuclear division	biological_process
GO:0005975	169/3434	406/11495	3.18E-06	0	carbohydrate metabolic process	biological_process
GO:0006261	92/3434	199/11495	3.32E-06	0	DNA-dependent DNA replication	biological_process
GO:0044282	95/3434	206/11495	3.33E-06	0	small molecule catabolic process	biological_process
GO:0000793	55/3434	107/11495	3.37E-06	0	condensed chromosome	cellular_component
GO:0000272	56/3434	107/11495	3.37E-06	0	polysaccharide catabolic process	biological_process
GO:0104004	101/3434	224/11495	4.18E-06	0	cellular response to environmental stimulus	biological_process
GO:0071214	101/3434	224/11495	4.18E-06	0	cellular response to abiotic stimulus	biological_process
GO:0016491	275/3434	667/11495	4.51E-06	0	oxidoreductase activity	molecular_function
GO:0034220	215/3434	542/11495	4.60E-06	0.002	ion transmembrane transport	biological_process
GO:0006260	93/3434	205/11495	4.75E-06	0.002	DNA replication	biological_process
GO:0005976	99/3434	221/11495	5.02E-06	0.002	polysaccharide metabolic process	biological_process
GO:0006310	93/3434	207/11495	5.04E-06	0.002	DNA recombination	biological_process
GO:0034312	16/3434	20/11495	5.12E-06	0.002	diol biosynthetic process	biological_process
GO:0034311	16/3434	20/11495	5.12E-06	0.002	diol metabolic process	biological_process
GO:0051186	148/3434	355/11495	5.17E-06	0.002	cofactor metabolic process	biological_process
GO:0098656	145/3434	346/11495	5.20E-06	0.002	anion transmembrane transport	biological_process
GO:0055114	332/3434	822/11495	5.25E-06	0.002	oxidation-reduction process	biological_process
GO:0006811	247/3434	641/11495	5.58E-06	0.004	ion transport	biological_process
GO:0044281	435/3434	1168/11495	6.07E-06	0.006	small molecule metabolic process	biological_process
GO:0022857	241/3434	620/11495	6.11E-06	0.006	transmembrane transporter activity	molecular_function

GO:0006855	108/3434	248/11495	6.22E-06	0.006	drug transmembrane transport	biological_process
GO:0044427	278/3434	738/11495	6.80E-06	0.006	chromosomal part	cellular_component

Supplementary Table S3 KEGG pathway enrichment analysis of differentially expressed genes.

**G-S VS P-S**							
layer1	layer2	layer3	ID	Input.number	Background.number	pvalue	FDR
1. Metabolism	1.10 Biosynthesis of other secondary metabolites	Phenylpropanoid biosynthesis	ko00940	10	13	0.000295	0.101523
1. Metabolism	1.0 Global and overview maps	Metabolic pathways	ko01100	277	857	0.000569	0.097806
1. Metabolism	1.6 Metabolism of other amino acids	Cyanoamino acid metabolism	ko00460	11	16	0.000691	0.079208
1. Metabolism	1.11 Xenobiotics biodegradation and metabolism	Metabolism of xenobiotics by cytochrome P450	ko00980	10	15	0.001759	0.100822
1. Metabolism	1.11 Xenobiotics biodegradation and metabolism	Polycyclic aromatic hydrocarbon degradation	ko00624	10	16	0.003538	0.15214
1. Metabolism	1.11 Xenobiotics biodegradation and metabolism	Dioxin degradation	ko00621	10	16	0.003538	0.135235
1. Metabolism	1.11 Xenobiotics biodegradation and metabolism	Naphthalene degradation	ko00626	11	19	0.005096	0.175306
1. Metabolism	1.7 Glycan biosynthesis and metabolism	Glycosphingolipid biosynthesis - globo and isoglobo series	ko00603	5	6	0.007243	0.226508
1. Metabolism	1.6 Metabolism of other amino acids	Taurine and hypotaurine metabolism	ko00430	5	6	0.007243	0.207632
1. Metabolism	1.11 Xenobiotics biodegradation and metabolism	Drug metabolism - cytochrome P450	ko00982	7	11	0.013102	0.346709

4. Cellular Processes	4.1 Transport and catabolism	Lysosome	ko04142	19	43	0.013619	0.334632
1. Metabolism	1.0 Global and overview maps	Degradation of aromatic compounds	ko01220	13	27	0.017529	0.402008
1. Metabolism	1.1 Carbohydrate metabolism	Glyoxylate and dicarboxylate metabolism	ko00630	12	25	0.022781	0.4898
1. Metabolism	1.6 Metabolism of other amino acids	Glutathione metabolism	ko00480	12	25	0.022781	0.460988
1. Metabolism	1.10 Biosynthesis of other secondary metabolites	Isoquinoline alkaloid biosynthesis	ko00950	9	17	0.023081	0.441105
1. Metabolism	1.10 Biosynthesis of other secondary metabolites	Betalain biosynthesis	ko00965	6	10	0.031241	0.565622
1. Metabolism	1.1 Carbohydrate metabolism	Propanoate metabolism	ko00640	8	16	0.046297	0.796312
1. Metabolism	1.0 Global and overview maps	Microbial metabolism in diverse environments	ko01120	61	190	0.088552	0.923083
5. Organismal Systems	5.8 Development and regeneration	Axon regeneration	ko04361	7	15	0.08857	0.896118
1. Metabolism	1.4 Nucleotide metabolism	Purine metabolism	ko00230	14	36	0.091663	0.900914
**M-F VS P-S**							
layer1	layer2	layer3	ID	Input.number	Background.number	pvalue	FDR
2. Genetic Information Processing	2.2 Translation	Ribosome	ko03010	77	91	8.16E-23	2.81E-20
1. Metabolism	1.2 Energy metabolism	Oxidative phosphorylation	ko00190	43	58	9.70E-10	1.67E-07
2. Genetic Information Processing	2.3 Folding, sorting and degradation	Proteasome	ko03050	23	32	2.00E-05	0.001379

5. Organismal Systems	5.10 Environmental adaptation	Thermogenesis	ko04714	45	81	9.54E-05	0.004104
2. Genetic Information Processing	2.4 Replication and repair	DNA replication	ko03030	23	38	0.001019	0.035065
1. Metabolism	1.1 Carbohydrate metabolism	Amino sugar and nucleotide sugar metabolism	ko00520	31	56	0.001228	0.038402
2. Genetic Information Processing	2.4 Replication and repair	Homologous recombination	ko03440	15	22	0.001422	0.040757
1. Metabolism	1.3 Lipid metabolism	Biosynthesis of unsaturated fatty acids	ko01040	8	10	0.004572	0.120971
1. Metabolism	1.0 Global and overview maps	Metabolic pathways	ko01100	333	857	0.004691	0.115264
4. Cellular Processes	4.2 Cell growth and death	Meiosis - yeast	ko04113	35	72	0.010386	0.223306
1. Metabolism	1.3 Lipid metabolism	Steroid biosynthesis	ko00100	11	17	0.011297	0.228606
1. Metabolism	1.0 Global and overview maps	Fatty acid metabolism	ko01212	15	26	0.01387	0.265066
1. Metabolism	1.0 Global and overview maps	Carbon metabolism	ko01200	49	108	0.014032	0.254054
5. Organismal Systems	5.6 Nervous system	Retrograde endocannabinoid signaling	ko04723	14	24	0.015283	0.262864
1. Metabolism	1.1 Carbohydrate metabolism	Glyoxylate and dicarboxylate metabolism	ko00630	14	25	0.023792	0.389731
1. Metabolism	1.1 Carbohydrate metabolism	Citrate cycle (TCA cycle)	ko00020	15	28	0.031449	0.491749
2. Genetic Information Processing	2.4 Replication and repair	Mismatch repair	ko03430	13	24	0.039799	0.595252

1. Metabolism	1.10 Biosynthesis of other secondary metabolites	Aflatoxin biosynthesis	ko00254	3	3	0.041977	0.601674
1. Metabolism	1.1 Carbohydrate metabolism	Pyruvate metabolism	ko00620	19	40	0.065217	0.89738
**M-F VS G-S**							
layer1	layer2	layer3	ID	Input.number	Background.number	pvalue	FDR
2. Genetic Information Processing	2.4 Replication and repair	DNA replication	ko03030	27	38	3.38E-07	0.000117
1. Metabolism	1.0 Global and overview maps	Metabolic pathways	ko01100	316	857	2.62E-05	0.004518
2. Genetic Information Processing	2.4 Replication and repair	Homologous recombination	ko03440	16	22	5.88E-05	0.006759
2. Genetic Information Processing	2.4 Replication and repair	Mismatch repair	ko03430	16	24	0.000296	0.025558
1. Metabolism	1.7 Glycan biosynthesis and metabolism	Glycosphingolipid biosynthesis - globo and isoglobo series	ko00603	6	6	0.000831	0.057326
4. Cellular Processes	4.2 Cell growth and death	Meiosis - yeast	ko04113	35	72	0.001038	0.059689
1. Metabolism	1.1 Carbohydrate metabolism	Amino sugar and nucleotide sugar metabolism	ko00520	28	56	0.001882	0.092742
2. Genetic Information Processing	2.4 Replication and repair	Base excision repair	ko03410	15	25	0.002251	0.097073

1. Metabolism	1.0 Global and overview maps	Microbial metabolism in diverse environments	ko01120	77	190	0.002281	0.087453
1. Metabolism	1.1 Carbohydrate metabolism	Starch and sucrose metabolism	ko00500	27	55	0.00315	0.108669
1. Metabolism	1.8 Metabolism of cofactors and vitamins	Ubiquinone and other terpenoid-quinone biosynthesis	ko00130	6	7	0.004289	0.134509
1. Metabolism	1.11 Xenobiotics biodegradation and metabolism	Metabolism of xenobiotics by cytochrome P450	ko00980	10	15	0.004352	0.125131
1. Metabolism	1.11 Xenobiotics biodegradation and metabolism	Aminobenzoate degradation	ko00627	10	15	0.004352	0.115505
1. Metabolism	1.1 Carbohydrate metabolism	Ascorbate and aldarate metabolism	ko00053	9	13	0.004743	0.116886
1. Metabolism	1.6 Metabolism of other amino acids	Glutathione metabolism	ko00480	14	25	0.007389	0.169946
1. Metabolism	1.6 Metabolism of other amino acids	Cyanoamino acid metabolism	ko00460	10	16	0.008433	0.181845
4. Cellular Processes	4.1 Transport and catabolism	Lysosome	ko04142	21	43	0.009401	0.190778
2. Genetic Information Processing	2.4 Replication and repair	Non-homologous end-joining	ko03450	7	10	0.012057	0.218929
1. Metabolism	1.6 Metabolism of other amino acids	Taurine and hypotaurine metabolism	ko00430	5	6	0.012115	0.208978
1. Metabolism	1.11 Xenobiotics biodegradation and metabolism	Naphthalene degradation	ko00626	11	19	0.012533	0.205892

Supplementary Table S4 Primers for qRT–PCR validation.

Gene Name	Gene ID	Primer-F	Primer-R
GH7 (Exoglucanase)	DQGG002368	CCAACAGGTACGACTCCGTC	CCATACCCTGGTCAGTGCAG
GH11 (endo-1,4-beta-xylanase)	DQGG004511	ATGGGAGCAGGTCAACAGTG	ATAGCTTGAGCACTGCCTGG
CE1 (acetyl xylan esterase)	DQGG010182	CCTCGTTCGCTCTGGGTATC	CGCCAAAAACGTTGGTCCAT
CE5 (acetylxylan esterase protein)	DQGG000985	GAGGCTATCAACTACCCCGC	CGCGTCGTCCATGATTTGAC
CBM5 (carbohydrate-binding module family 5 protein)	DQGG006344	GGACGGCTAAATGGTGGTCA	TGCGGACGGATGTTGTGTTA
AA9-1 (Auxilliary Activities Family 9)	DQGG009829	CTGGACCTCCACCATCCCTA	GAGCTCAGCTGAAGTGGGTT
AA9-2 (glycoside hydrolase family 61 protein)	DQGG009624	CGAAGCCTACCACTTCGTCA	TGGGCACATGTTGTAGGTCC
GT2 (glycosyltransferase family 2 protein)	DQGG006597	TCCGCATGATCGTTCTCCAG	GTTGCTGACGAGACGAGGAA
PL14 (polysaccharide lyase family 14 protein)	DQGG010936	GTGTCGATTTGACCACTGCG	CTGTCGCCTCCGTAGAATCC
Heat shock protein 60	DQGG011020	TGTGAAACTGTTGGGTGCCT	TCCGAAAGTTTGGCGATCCA
HSP90-domain-containing protein	DQGG001016	GCGAGACACTGCAATCGAAC	ACCGAGCCGAAAGTCTCTTG
Carbonic anhydrase1 (CA-1)	DQGG004716	ACCTCTCAGCTCGTGAAAGC	CGACGAGAGCACGATAACCA
Carbonic anhydrase 2 (CA-2)	DQGG008753	GCGCAACAAGCTTAAGGGTC	AATAGGGCCACCTTCAGCAC
cAMP-dependent protein kinase PKA1	DQGG004293	ATCAAAACCTTCCGTCGCCT	GATAAGTCCCCTTTGGCCGT
cAMP-dependent protein kinase PKA2	DQGG007322	AGCCCAAAGTGCTCGTACTC	CCCCTGGACGGTCATCAAAA

Transcriptional factor Myb	DQGG003605	CCGCAATTGATCGAACCACC	TGTACGGCCGGGAAAGTAAG
Transcription factor steA	DQGG001965	CTCTGGCCAAGTCGATACCC	GTAGTTGCCCTGAGCATCGT
18s	DQGG007047	TTGAACTTCGTGGAGTCGGG	TTAAACCGTACCCAGGCGAC
